# Searching for plant-derived antivirals against dengue virus and Zika virus

**DOI:** 10.1186/s12985-022-01751-z

**Published:** 2022-02-22

**Authors:** Emerson de Castro Barbosa, Tânia Maria Almeida Alves, Markus Kohlhoff, Soraya Torres Gaze Jangola, Douglas Eduardo Valente Pires, Anna Carolina Cançado Figueiredo, Érica Alessandra Rocha Alves, Carlos Eduardo Calzavara-Silva, Marcos Sobral, Erna Geessien Kroon, Luiz Henrique Rosa, Carlos Leomar Zani, Jaquelline Germano de Oliveira

**Affiliations:** 1 Instituto René Rachou - Fiocruz Minas, Fundação Oswaldo Cruz, Av. Augusto de Lima 1715, Belo Horizonte, Minas Gerais 30190-002 Brasil; 2 School of Computing and Information Systems, University of Melbourne, Melbourne, VIC 3052 Australia; 3 Departamento de Ciências Naturais, Universidade Federal de São João del-Rei, Campus Dom Bosco - Praça Dom Helvécio, 74, São João del-Rei, Minas Gerais 36301-160 Brasil; 4 Departamento de Microbiologia, Instituto de Ciências Biológicas, Universidade Federal de Minas Gerais, Av Antônio Carlos 6627, Belo Horizonte, Minas Gerais 31270-901 Brasil

**Keywords:** Antiviral, Dengue virus, Zika virus, Alkaloids, Natural products, Plant extract, *Hippeastrum*, Virucidal, Bioactivity-guided fractionation, Compounds

## Abstract

**Background:**

The worldwide epidemics of diseases as dengue and Zika have triggered an intense effort to repurpose drugs and search for novel antivirals to treat patients as no approved drugs for these diseases are currently available. Our aim was to screen plant-derived extracts to identify and isolate compounds with antiviral properties against dengue virus (DENV) and Zika virus (ZIKV).

**Methods:**

Seven thousand plant extracts were screened in vitro for their antiviral properties against DENV-2 and ZIKV by their viral cytopathic effect reduction followed by the 3-(4,5-dimethylthiazol-2-yl)-2,5-diphenyltetrazolium bromide (MTT) method, previously validated for this purpose. Selected extracts were submitted to bioactivity-guided fractionation using high- and ultrahigh-pressure liquid chromatography. In parallel, high-resolution mass spectrometric data (MSn) were collected from each fraction, allowing compounds into the active fractions to be tracked in subsequent fractionation procedures. The virucidal activity of extracts and compounds was assessed by using the plaque reduction assay. EC_50_ and CC_50_ were determined by dose response experiments, and the ratio (EC_50_/CC_50_) was used as a selectivity index (SI) to measure the antiviral *vs.* cytotoxic activity. Purified compounds were used in nuclear magnetic resonance spectroscopy to identify their chemical structures. Two compounds were associated in different proportions and submitted to bioassays against both viruses to investigate possible synergy. In silico prediction of the pharmacokinetic and toxicity (ADMET) properties of the antiviral compounds were calculated using the pkCSM platform.

**Results:**

We detected antiviral activity against DENV-2 and ZIKV in 21 extracts obtained from 15 plant species. *Hippeastrum* (Amaryllidaceae) was the most represented genus, affording seven active extracts. Bioactivity-guided fractionation of several extracts led to the purification of lycorine, pretazettine, narciclasine, and narciclasine-4-O-β-D-xylopyranoside (NXP). Another 16 compounds were identified in active fractions. Association of lycorine and pretazettine did not improve their antiviral activity against DENV-2 and neither to ZIKV. ADMET prediction suggested that these four compounds may have a good metabolism and no mutagenic toxicity. Predicted oral absorption, distribution, and excretion parameters of lycorine and pretazettine indicate them as candidates to be tested in animal models.

**Conclusions:**

Our results showed that plant extracts, especially those from the *Hippeastrum* genus, can be a valuable source of antiviral compounds against ZIKV and DENV-2. The majority of compounds identified have never been previously described for their activity against ZIKV and other viruses.

**Supplementary Information:**

The online version contains supplementary material available at 10.1186/s12985-022-01751-z.

## Introduction

*Zika virus* (ZIKV) and *Dengue virus* (DENV), members of the *Flaviviridae* family, are arboviruses of great importance in human Public Health worldwide. Primarily asymptomatic, ZIKV and DENV infected patients may have mild clinical symptoms such as fever, headache, and rashes. However, ZIKV can also cause severe neurologic manifestations in fetuses and newborns after congenital infection and Guillain-Barré syndrome in adults [1–3]. There are no currently approved vaccines for ZIKV infection [4]. This scenario is worsened by the imminent risk of DENV infections, which, although it already has a licensed vaccine, CYD-TDV (Dengvaxia), its indication is not well established. CYD-TDV efficacy varies according to DENV serotype, serostatus (with detectable dengue antibodies), and age [5, 6]. According to the World Health Organization (WHO), Dengvaxia is recommended for people living in regions where dengue is endemic, from 9 to 45 years old, and who have had at least one previous DENV infection [5]. Unfortunately, there are an estimated 100–400 million dengue infections each year, and severe dengue is a potentially deadly complication due to plasma leaking, fluid accumulation, respiratory distress, severe bleeding, or organ impairment. Furthermore, without proper medical care, severe dengue can reach up to 10% case fatality [5, 7, 8]. As for Zika, there are no approved antivirals for dengue treatment. Treatments are palliative and mainly directed toward alleviating symptoms, such as fever and rash, by administering antipyretics, antihistamine, and fluids against dehydration [9–11]. Therefore, Zika and dengue outbreaks have been triggered intense efforts to drug repurposing and identification of novel antiviral agents for specific treatment [12, 13]. Different approaches and methodologies have been used for drug repurposing to find antivirals against ZIKV and DENV, such as testing specific compounds with known antiviral activity in other virus models and screening of libraries composed of hundreds of bioactive molecules, many of them already approved for human use. These molecules target viral and cellular components, including nucleosides analogues, nucleoside synthesis inhibitors, drugs targeting viral enzymes, anticancer and anti-inflammatory molecules, antibiotics, antiparasitics, among others [11, 14].

Compounds derived from plants have long been used worldwide as a source of medicines since ancient times until today [15, 16]. Indeed, natural products derived from plants and other living organisms represent a huge reservoir of bioactive chemical diversity and have been successful sources of potential drug leads to new therapeutic agents for many human diseases, including those caused by viruses [17–19]. Our strategy was to screen large numbers of plant extracts for their anti-flavivirus activities to identify compounds that exhibit antiviral activity against DENV and ZIKV. Other studies have shown that higher plants represent a recognized source of antiviral drug leads [19, 20]. Thus, the aim of our study was threefold: (1) to identify extracts of plants collected in distinct Brazilian biomes as potential sources of antiviral compounds; (2) to identify and purify the active compounds present in the selected extracts and (3) assess the antiviral activity of the purified compounds against DENV-2 and ZIKV.

## Methods

### Reagents

Solvents and reagents used in analytical methods were purchased from Merck (Brazil). 3-(4,5-dimethylthiazol-2-yl)-2,5-diphenyl tetrazolium bromide (MTT), cell culture media, and supplements were purchased from Thermo (USA). Trypsin–EDTA and carboxymethyl cellulose were purchased from Sigma-Aldrich (USA). Formic acid (> 98%) was purchased from Fluka (USA). Lycorine (HCl Salt Form) was purchased from BOC Sciences (USA).

### Cells, viruses, and Interferon-α 2B

C6/36 (ATCC #CRL-1660) cells derived from larvae of *Aedes albopictus* mosquito were used to obtain working stock of DENV-2 and ZIKV. Baby Hamster Kidney 21 cells (BHK-21; ATCC #CCL-10) and Vero cells (ATCC #CCL-81) were used, respectively, for virus titration and antiviral assays activity against DENV-2 and ZIKV. The ZIKV used in this study (PE243/2015; GenBank: KX197192.1) was kindly given by Dr. Marli Tenório Cordeiro from Aggeu Magalhães Institute, Fiocruz Pernambuco, Brazil. The working stock of low passage ZIKV (7.4 × 10^8^ PFU/ml) was prepared and stored at − 80 °C in our lab at the René Rachou Institute, Fiocruz Minas, Brazil. ZIKV stock titration was performed by plaque formation assay in Vero cells. The DENV-2 sample was provided by Dr. Luiz Tadeu Figueiredo from University of São Paulo, Ribeirão Preto, Brazil. DENV-2 was multiplied in C6/36 cells and titrated by plaque formation assay in BHK-21 cells. Interferon-α 2B (INREC, Uruguay) was used as a positive control in antiviral assays.

### Plant material

Plant specimens were collected in different Brazilian biomes by Dr. Marcos Sobral, from Universidade Federal de São João del-Rei/MG. *Hippeastrum glaucescens (H. glaucescens)* and *Hippeastrum puniceum* (*H. puniceum*) were also collected by Carlos Alberto Ferreira Junior at Fundação Zoobotânica de Belo Horizonte-MG. Both researchers were also responsible for the identification of the plant specimens. Voucher codes are shown in Table 1.

**Table 1:** Plant extracts with anti-DENV-2 and anti-ZIKV activity: EC_50_, CC_50_, SI and virucidal activity

Family	Plant species	Voucher	Part	Extract	EC_50_ DENV-2	CC_50_ BHK-21	SI	Virucidal activity	EC_50_ ZIKV	CC_50_ Vero	SI	Virucidal activity
*Amaryllidaceae*	*Hippeastrum glaucescens*	HUFSJ-2914	Flower	13356	27.9	> 100	> 3.6	No	56.8	> 100	> 1.8	No
Amaryllidaceae	*Hippeastrum glaucescens*	HUFSJ-2914	Bulb	13358	3.9	8.6	2.2	No	8.9	53.0	5.9	No
Amaryllidaceae	*Hippeastrum* sp.	HUFSJ-3007	Root	13418	27.5	76.2	2.8	No	41.8	> 100	> 2.4	No
Amaryllidaceae	*Hippeastrum glaucescens*	HUFSJ-4494	Bulb	17006	6.2	8.4	1.4	No	10.6	44.1	4.2	No
Amaryllidaceae	*Hippeastrum puniceum*	BHZB-12069	Flower	17007	3.1	8.7	2.8	No	6.8	18.2	2.8	No
Amaryllidaceae	*Hippeastrum puniceum*	BHZB-12069	Root	17010	3.1	5.9	1.9	No	5.5	16.5	3.0	No
Amaryllidaceae	*Hippeastrum puniceum*	BHZB-12069	Bulb	17011	0.8	1.3	1.7	No	1.1	4.2	3.8	No
Annonaceae	*Duguetia furfuracea*	HUFSJ-2345	Fruit	12829	9.8	> 100	> 10.2	+	34.7	> 100	> 2.9	+
Asteraceae	*Chrysolaena* sp.	HUFSJ-2686	Flower	13211	8.9	16.4	1.8	+	35.1	> 100	> 2.8	+
Erythroxylaceae	*Erythroxylum* sp.	HUFSJ-2580	Stem	13023	9.1	41.1	4.5	+	29.3	> 100	> 3.4	+
Fabaceae	*Chamaecrista* sp.	HUFSJ-2193	Fruit	11279	2.9	35.4	11.9	+	53.7	> 100	> 1.9	+
Fabaceae	*Chamaecrista sp*.	HUFSJ-2193	Stem	11280	7.8	> 100	> 20.5	No	> 100	> 100	> 1.0	No
Malpighiaceae	*Banisteriopsis argyrophylla*	HUFSJ-2201	Stem	11298	12.5	36.6	2.9	+	48.9	> 100	> 2.0	+
Malpighiaceae	*Banisteriopsis* sp.	HUFSJ-1995	Stem	11117	34.8	80.5	2.3	+	63.2	> 100	> 1.6	+
Malpighiaceae	*Byrsonima coccolobifolia*	HUFSJ-2683	Flower	13203	9.4	26.7	2.8	+	43.4	> 100	> 2.3	+
Primulaceae	*Ardisia* sp.	HUFSJ-2368	Stem	12875	11.8	31.1	2.6	+	43.9	> 100	> 2.3	+
Rubiaceae	*Palicourea* sp.	HUFSJ-2360	Flower	12860	16.5	> 100	> 6.0	+	62.4	> 100	> 1.6	+
Sapindaceae	*Urvillea* sp.	HUFSJ-1997	Stem	11123	23.8	31.5	1.3	+	43.1	> 100	> 2.3	+
Sapindaceae	*Cupania* sp.	HUFSJ-1992	Leaf	11107	19.5	38.7	2.0	+	52.7	> 100	> 1.9	+
Sapindaceae	*Cupania sp*.	HUFSJ-1992	Stem	11108	17.8	47.9	2.7	+	21.6	> 100	> 4.6	+
Ochnaceae	*Ouratea* sp.	HUFSJ-2555	Flower	12969	5.2	51.7	9.9	+	26.2	> 100	> 3.8	+

EC_50_: Half maximum effective concentration; CC_50_: Half maximum cytotoxic concentration; SI: Selectivity Index = ratio CC_50_/EC_50_; ( +) positive virucidal activity

### Preparation of extracts

In brief, fresh plant parts were separated, cut into small pieces, and approximately 20 g placed in a 50 ml flask. The flask was then filled with absolute ethanol (Merck, Brazil) and kept at room temperature in the dark for at least one week. The extract was filtered and dried under vacuum at 45 °C. Stock solutions of the extracts at 20 mg/ml were prepared using 90% dimethyl sulfoxide (DMSO) and stored at –20 °C. For isolation of active compounds, about 2 kg of *H. puniceum* bulbs were used.

### Validation of the MTT method for antiviral screening against ZIKV and DENV-2

The validation of the MTT method [21–23] for High Throughput Screening (HTS) of antivirals against ZIKV and DENV was performed in accordance with the Assay Guidance Manual published by the National Institutes of Health (NIH) [24]. Briefly, tests were made with three levels of signals as follows: high signal (H) for cell control, medium signal (M) for antiviral control and, low signal (L) for virus infection control. the MTT assays were done in sets of 96-well plates containing positive and negative controls (Additional file 1: Fig. S1). For the assays with ZIKV and DENV-2, 1.0 × 10^4^ Vero cells per well and 8.0 × 10^3^ BHK-21 cells per well, respectively, were seeded in 96-well plates and incubated for 24 h in MEM with 5% FBS. We used 200 µL of MEM with 0.25% DMSO for the high signal wells (H); 100 µL of ZIKV or DENV-2 suspension at m.o.i. of 2 and 100 µL of IFN-α 2b (100 IU/ml) for the medium signal (M); and for low signal (L), 100 µL of ZIKV or DENV-2 suspension at m.o.i. of 2 were added 100 µl of MEM medium. After three days post-infection (p.i.) with DENV-2 and 4 days p.i. with ZIKV, the reduction of viral CPE was verified by optic microscopy using a scoring system as described [23] and by the MTT method. The evaluation of the test and the variability between wells and between plates was done using the software available at the NIH website [24].

### Screening of antiviral activity by cytopathic effect reduction and by the MTT method

Plant extracts were screened for their cytotoxic and anti-ZIKV and anti-DENV activities in Vero cells and BHK-21, respectively, in 96-well culture plates by observing the reduction of the viral cytopathic effect (CPE) [23] and then measured by the MTT method [21, 22]. The crude extracts were dissolved in 4% DMSO aqueous solution, and their concentration was normalized to 50 µg/ml with Eagle's minimum essential medium (MEM) supplemented with 2% fetal bovine serum (FBS). Briefly, 8 × 10^3^ of BHK-21 cells or 1 × 10^4^ of Vero cells per well were seeded in a 96-well plate and incubated for 24 h at 37 °C and 5% CO_2_ atmosphere. Extract solution and virus suspension, at a multiplicity of infection (m.o.i.) of 2, for both viruses, were simultaneously added on 70% confluent monolayers. Each extract was tested in duplicate. Controls of untreated infected and untreated uninfected cells incubated with or without DMSO were included. Interferon-α 2B, in concentrations of 500 and 1000 IU/mL, was used as a positive antiviral control in all antiviral assays. After three days post-infection (p.i.) with DENV-2 and 4 days p.i. with ZIKV, the reduction of viral CPE was verified by optic microscopy using a scoring system as described by Kudi and Myint (1999) [23] and by the MTT method. Briefly, the supernatants were removed, and 30 µl of a stock solution of MTT (2 mg/ml) dissolved in phosphate-buffered saline were added to each well. After 90 min of incubation at 37 °C, 130 µl of DMSO were added to each well, and plates were homogenized for 5 min at 500 rpm. The absorbance values of each reaction were measured in an ELISA reader (Spectra Max, Molecular Devices, USA) at λ_540_ nm. Extracts were considered active when they showed at least 50% of protection or reduction of the viral CPE by at least 50%. Cytotoxicity (CC_50_) and anti-ZIKV/DENV-2 (CE_50_) experiments were performed in duplicate and repeated at least three times.

### Determination of CC_50_

The half-maximal cytotoxic concentration (CC_50_) was calculated as the extract/compound concentration at which only 50% of cells were alive, in comparison with non-treated cell controls. Compounds and selected extracts that tested positive for anti-ZIKV or anti-DENV-2 activity at the screening step had their CC_50_ determined by using an 8‐point dose range in 96 well microplate containing  Vero or BHK-21 cell monolayers. Cell viability was observed by optic microscopy and measured by the MTT method as described. The experiments were performed in duplicate and repeated at least three times.

### Determination of EC_50_

Viral CPE and cell viability were observed by optic microscopy and measured by the MTT method as previously described. The half-maximal effective concentration (EC_50_) was calculated as the extract/compound concentration at which only 50% of cells were alive, in comparison with non-infected cell controls. Compounds and extracts that tested positive for anti-ZIKV or anti-DENV-2 activity at the screening step had their EC_50_ determined using an 8‐point dose range in 96 well microplates in Vero cells or BHK-21 monolayers, respectively. The experiments were performed in duplicate and repeated at least three times.

### Virucidal mechanism of action

The virucidal activity of extracts and compounds against ZIKV and DENV was assessed by plaque reduction assay [25] in Vero and BHK-21 cells, respectively, in 24 well microplates. Prior viral adsorption, equal volumes of viral suspension and extract or compound dilutions, corresponding to its EC_50_, EC_50_/2, and two times EC_50,_ were mixed and incubated for 60 min at 37 °C and 5% CO_2_ atmosphere_._ Virus suspension without the extract/compound was included as a control. Surviving infectious viral particles were assessed by viral plaque formation assay. Briefly, the infected monolayers were overlaid with 1.5% carboxymethyl cellulose containing MEM with 2% FBS and incubated for five days. After that, cell monolayers were fixed with 10% formaldehyde and stained with 1% crystal violet 1% in an 30% ethanol aqueous solution. Extract and compounds were considered virucidal when their EC_50_ reduced at least by 90% the number of virus plaques compared to the untreated virus control [26, 27].

### Bioassay-guided chromatographic fractionation

Selected extracts were fractionated in a Nexera UHPLC-system (Shimadzu, Japan) hyphenated to a MaXis ETD high-resolution ESI-QTOF mass spectrometer (Bruker, Germany) controlled by the Compass 1.7 software package (Bruker, Germany). Samples were diluted to final 5 mg/ml, and 1–3 µl (5–15 µg) injected on a Shim-Pack XR-ODS-III column (C18, 2.2 µm, 2.0 × 150 mm) (Shimadzu, Japan) at 40 °C under a flow rate of 400 μl/min. An additional identical run was performed with injection of 10 μl (50 µg). Instead of being sent to the mass spectrometer, the column effluent was collected in 80 wells of a polypropylene microtiter plate (200 µl/well/fraction). The mobile phases A and B (0.1% formic acid in distilled water and acetonitrile, respectively) formed an eluent gradient of initial 5 min 5% B, a linear gradient to 100% B in 40 min, and hold at 100% B for 5 min. The mass spectra were acquired in positive mode at a spectra rate of 2–5 Hz. Ion-source parameters were set to 500 V endplate offset, 4500 V capillary voltage, 3.0 bar nebulizer pressure, 8 l/min, and 200 °C dry gas flow and temperature, respectively. Data-dependent fragment spectra were recorded using a collision energy range between 15 and 60 eV. Ion cooler settings were optimized for an m/z 40–1000 range using a calibrant solution of 1 mM sodium formate in 50% 2-propanol. Mass calibration was achieved by initial ion-source infusion of 20 µl calibrant solution and post-acquisition recalibration of the raw data. Compound detection was performed by chromatographic peak dissection with subsequent formula determination according to exact mass and isotope pattern (MS1). Putative identification was based on the comparison of compound fragment spectra (MS2) with reference spectra of an in-house database of standard compounds, the public spectra database MassBank [28] as well as in silico fragment spectra generated from the Universal Natural Product Database (UNPD-ISDB) [29]. The collected fractions were dried in a vacuum centrifuge at 45 °C before being used in the bioassays. Compounds in the active fractions were identified by their retention time, exact m/z, and MS/MS fragmentation. These features were then used to track these compounds during the extract's preparative fractionation steps, aiming to isolate the active compounds for structural elucidation and further bioassays. The antiviral activity of all fractions was accessed using the bioassays with DENV-2 or ZIKV by the MTT method as described. IFN alpha 2B and the original crude extract were positive controls for antiviral activity.

### Nuclear magnetic resonance analysis

Mono and bidimensional 1H and inversed detection 13C NMR experiments were run on a Bruker Avance 400 MHz spectrometer (Bruker Daltonics, Germany) using TMS as an internal standard. Samples were dissolved in chloroform-d. The experiments were run at 27 °C. Data were analysed using TopSpin software from Bruker.

### Synthesis of Lycorine mono and di-acetylated derivatives

The methodology described by Wang et al. [30] was used. The chemical structures of the mono and di-acetylated derivatives were confirmed by comparing their MS and NMR spectral data with those described by the mentioned authors. The compounds were dissolved in DMSO and assayed at 0.5% DMSO final concentration.

### Investigation of pretazettine and lycorine synergism against DENV-2 and ZIKV

The antiviral effect of the association of lycorine and pretazettine against DENV-2 and ZIKV was evaluated in vitro by a modified isobologram method [31, 32]. The results were analysed by nonlinear regression in the GraphPad Prism 5 software (GraphPad Software, Inc.). The EC_50_ and CC_50_ of each combination were assessed by the MTT method as described previously. Thus, two EC_50_ and CC_50_ for each of the five combination curves were calculated separately using the known concentration ratios of lycorine and pretazettine. The fractional inhibitory concentration of lycorine (FIC_L_) and pretazettine (FIC_P_) were calculated for each point, i.e. [EC_50_ when in combination]/[EC_50_ of drug alone]. The sum of the FIC (ΣFIC) with the equation ΣFIC = FIC lycorine + FIC pretazettine was calculated [33]. From the median of the sum of all FIC (*x*ΣFIC), the fractional inhibitory concentration index (FICI) value was obtained and used to classify the interaction between lycorine and pretazettine. There would be synergy when *x*ΣFIC ≤ 0.5; indifference when *x*ΣFIC between 0.5 and ≤ 4 or antagonism if *x*ΣFIC was ≥ 4 [33]. Isobolograms were built by plotting the FIC of lycorine vs. pretazettine. The ratio reveals the effect of their association on toxicity and antiviral activity [31, 32, 34, 35].

### ADMET prediction

Pharmacokinetics and toxicity properties of compounds were predicted using the pkCSM platform [36]. pkCSM is a comprehensive web-based, user-friendly resource that allows for the rapid screening of 30 different properties regarding absorption, distribution, metabolism, excretion, and toxicity (http://biosig.unimelb.edu.au/pkcsm). pkCSM relies on the concept of graph-based signatures [37, 38] to model small molecule physicochemical and geometrical properties. These are used as evidence to train machine learning-based models, which have been invaluable to predict and optimize ligand pharmacokinetic properties [39]. Compounds structures were provided to the pkCSM web server as their canonical SMILES representation.

### Data analysis and selectivity index

Bioassay data were normalized to untreated uninfected cells and the EC_50_ and CC_50_ values were obtained using GraphPad Prism 5 based on nonlinear logistic regression of the experimental dose–response curves. The values correspond to three independent assays' average and standard deviation with at least 8 sample distinct concentrations. The CC_50_ and EC_50_ values ratio was used as a selectivity index (SI). The extracts and compounds with higher SI values were considered the most promising and selected for further investigation if available in sufficient quantities.

## Results

### The MTT method is a reliable tool for antiviral high-throughput screening (HTS) against DENV and ZIKV

The results of the validation of the MTT method for antiviral HTS against ZIKV in Vero cells and DENV-2 in BHK-21 cells are shown in Additional file 1: Figs. S2 and S3. We observed no effects of systematic errors, which could be verified if the difference of over 20% in the values of each column or observing the distribution of points (each point is equivalent to the reading of a well). We noticed a considerable edge effect in both ZIKV and DENV assays. Indeed, edge effects are usually due to evaporation in the wells on the sides of the plate, especially when the assay requires incubation for very long periods. To reduce this variability, the incubation time was reduced from 96 to 72 h. Finally, for the assessment of the MTT assay and the variability between plates, the acceptance criterion is that the L or MIN signal (infected and untreated cells) should not show any significant change. Furthermore, the coefficient of variation between the H or MAX signals (uninfected and untreated cells) and between the M or MED signals (infected cells and treated with the positive control) must not exceed 20%, and the Z' factor must be above of 0.4 (data not shown). All criteria listed in the statistical analysis were met. Finally, it is worth mentioning that we demonstrated that the CE_50_ values of several extracts obtained from the assessment of viral CPE protection against DENV-2 and ZIKV were similar to those obtained by the MTT assay (data not shown).

### Antiviral, EC_50_, CC_50_ , and virucidal activities of crude plant extracts

A total of 7,000 extracts obtained from plants occurring in different Brazilian biomes were screened at 20 µg/ml on cells infected with DENV-2. A smaller number (1,000 extracts) were also tested against ZIKV. As a result, 21 plant extracts from ten plant families displayed consistently in vitro activity against DENV-2 and ZIKV, and their EC_50_, CC_50_, and virucidal properties were determined (Table 1). Among the 21 selected extracts, 12 exhibited good DENV-2 activity, disclosing EC_50_ values < 10 µg/ml, while nine had a moderate activity with an EC_50_ ranging from 10 to 35 µg/ml. Four extracts exhibited good anti-ZIKV activity and showed EC_50_ values < 10 µg/ml, while 17 had a moderate anti-ZIKV activity with an EC_50_ ranging from 10 to 100 µg/ml. The extracts of the Amaryllidaceae family were cytotoxic in very low concentrations, with CC_50_ values < 10 µg/ml, while extracts from other families presented CC_50_ > 10 µg/ml. The SI values of the extracts ranged from 1.3 to > 20.5 µg/ml for DENV-2 and 1.0 to 5.9 for ZIKV. Afterward, inhibition of infection due to virucidal effect on DENV and ZIKV particles was tested using at least three non-cytotoxic concentrations. Thirteen extracts were considered virucidal since they caused a 90% or higher reduction in plaque numbers (Table 1).

### Bioassay-guided fractionation

The choice of extracts for bioassay-guided fractionation was based on their antiviral activity and sample availability. Thirteen active extracts, eight virucidal and five non-virucidal (listed in Tables 1, 2) were subjected to bioactivity-guided fractionation to identify their antiviral compounds. The extracts from fruits of *Duguetia furfuracea* (Annonaceae), stems of *Banisteriopsis argyrophylla* (Malpighiaceae), and flowers of *Palicourea* sp. (Rubiaceae) did not yield fractions with significant activity against ZIKV and DENV-2 (Table 2). The flower from *Ouratea* sp. (Ochnaceae) afforded an extract for which the activity appeared only in the first experiment. The fractionation of this extract yielded highly polar fractions of the UHPLC RP-18 chromatography; however, no useful MS or MS/MS spectra could be obtained. In the active fractions of stem extract of *Chamaecrista* sp. (Fabaceae) the presence of agarobiose and leuhistin was proposed based on the HRMS/MS data. Similarly, fragment spectra indicated the presence of leuhistin and plumbagin in the active fractions of its fruit extracts. Extracts of *H. glaucescens* (Amaryllidaceae) afforded several active fractions, from which HRMS/MS data suggested the presence of pancratine (flowers), pretazettine (flowers and bulbs), rutine (flower), lycorine (flowers and bulbs), pseudolycorine, pancracine and nangustine (bulbs) (Table 2). HRMS/MS spectra of the active fractions from the extracts of *H. puniceum* suggested the presence of incartine and pseudolycorine from its flowers. The MS/MS spectra of root extract were compatible with lycoramine, narcissidine acetate, and 6-hydroxy-hippeastrine. narciclasine, kalbreclasine, NXP, pseudolycorine, lycoranine E, lycoranine C, N-norlycoramine, incartine, lycorine, crisarnine, acetylnerbowdine, and narcissidine acetate were detected in the bulbs extract of *H. puniceum* (Table 2). Lycorine, narciclasine, and NXP were isolated, and their identity was confirmed based on their NMR spectra or by comparison with a commercial standard. Pretazettine, as well, was compared with 1H NMR data from those described by Moraes [40]. Commercial rutine was compared with the MS/MS spectra of its respective flower extract fraction of *H. glaucescens.*

**Table 2:** Compounds identified by UHPLC-HRMS/MS in active extract fractions against DENV-2 and ZIKV

Extract number	Plant family	Species	Part	Chemical compound
13356		*Hippeastrum glaucescens*	Flower	Pancratine, pretazettine, rutine and lycorine
13358	*Amaryllidaceae*	*Hippeastrum glaucescens*	Bulb	Lycorine and pretazettine
13418	*Amaryllidaceae*	*Hippeastrum* sp.	Root	Lycorine
17006	* Amaryllidaceae*	*Hippeastrum glaucescens*	Bulb	Lycorine, pseudolycorine, pancracine, nangustine and pretazettine
17007	*Amaryllidaceae*	*Hippeastrum puniceum*	Flower	Pseudolycorine, incartine
17010	*Amaryllidaceae*	*Hippeastrum puniceum*	Root	Lycorine, lycoramine, narcissidine acetate and 6-hydroxy-hippeastrine
17011	*Amaryllidaceae*	*Hippeastrum puniceum*	Bulb	Narcissidine acetate, narciclasine, lycorine, kalbreclasine, lycoranine E, lycoranine C, acetylnerbowdine, incartine, crisarnine, pseudolycorine, N-norlycoramine and narciclasine-4-*O*-*β*-D-xylopyranoside (NXP*)*
12829	*Annonaceae*	*Duguetia furfuracea*	Fruit	No active fractions
11280	*Fabaceae*	*Chamaecrista* sp.	Stem	Agarobiose and leuhistin
11279	*Fabaceae*	*Chamaecrista* sp.	Fruit	Leuhistin and plumbagin
11298	*Malpighiaceae*	*Banisteriopsis argyrophylla*	Stem	No active fractions
12860	*Rubiaceae*	*Palicourea* sp.	Flower	No active fractions
12969	*Ochnaceae*	*Ouratea* sp.	Flower	Mix of polar or ionizable compounds

### Anti-DENV-2 and anti-ZIKV activity of rutine, lycorine and its derivatives, pretazettine, narciclasine and NXP

Among the 22 compounds detected in the bioactive fractions, three were isolated and identified: pretazettine, narciclasine, and NXP. Rutine and lycorine were acquired from commercial sources. Lycorine derivatives were synthesized in our lab. These compounds were tested for their antiviral and cytotoxic properties. Rutine showed low toxicity against BHK-21 and Vero cells, with CC_50_ of > 300 µM and no antiviral activity against DENV-2 and ZIKV (Table 3). Pretazettine showed antiviral activity against DENV-2 (EC_50_ of 0.8 µM and SI of 6.8) and ZIKV (EC_50_ of 1.9 µM and SI of 3.8) with CC_50_ of 5.4 µM for BHK-21 cells and 7.2 µM for Vero cells. Narciclasine, isolated from *H. puniceum*, showed anti-DENV and anti-ZIKV activity at very low concentrations, with EC_50_ of 0.02 µM for both viruses, but also a CC_50_ in the low micromolar range (0.09 µM for BHK-21 and 0.12 µM for Vero cells), resulting in SI values of 4.5 and 6.0 for DENV-2 and ZIKV, respectively. Its sugar conjugate, narciclasine-4-*O*-*β*-D-xylopyranoside or NXP, described recently by Katoch et al. [41], was much less active against these viruses (EC_50_ = 7.9 µM) and less toxic to BHK-21 and Vero cells (CC_50_ of 39.3 µM and 51.8 µM, respectively) (Table 3). Lycorine was active against DENV-2 (EC_50_ of 0.5 µM and SI of 8.6) and ZIKV (EC_50_ of 0.9 µM and SI of 3.8). Similarly, to other *Amarillydaceae* alkaloids, lycorine was toxic at low concentrations to BHK-21 and Vero cells, with CC_50_ of 4.3 µM and 3.4 µM, respectively. Using commercial lycorine, we synthesized 1-acetyl-lycorine and 1,2-diacetyl-lycorine derivatives to test them against DENV-2 and ZIKV. 1,2-diacetyl-lycorine showed CC_50_ > 100 µM on BHK-21 and Vero cells but was inactive against DENV-2 and ZIKV. Likewise, the 1-acetyl-lycorine showed decreased cytotoxicity in BHK-21 cells (CC_50_ of 7.8 µM) and in Vero cells (CC_50_ of 19.8 µM) when compared to lycorine. However, 1-acetyl-lycorine was inactive against DENV-2 and showed very weak activity against ZIKV, with EC_50_ of 19.3 µM and SI of 1.0. The CC_50_ and EC_50_ titration curves of lycorine, pretazettine, narciclasine, against ZIKV and DENV are shown in the Additional file 1: Fig. S3.

**Table 3:** Antiviral activities of compounds against DENV-2 and ZIKV

Compounds	DENV-2			ZIKV			CC_50_ (µM)	
	EC_50_ (µM)	Virucidal activity	SI	EC_50_ (µM)	Virucidal activity	SI	BHK-21	Vero
Lycorine	0.5	No	8.6	0.9	No	3.8	4.3	3.4
Rutine	Inactive	No	–	Inactive	NT	–	> 300	> 300
Pretazettine	0.8	No	6.8	1.9	No	3.8	5.4	7.2
Narciclasine	0.02	No	4.5	0.02	No	6.0	0.09	0.12
Narciclasine-4-*O*-*β*-D-xylopyranoside	7.9	No	4.9	7.9	No	6.5	39.3	51.8
1-acetyl-lycorine*	Inactive	No	–	19.3	No	1.0	7.8	19.8
1,2-diacetyl-lycorine*	Inactive	No	–	Inactive	No	–	> 100	> 100

EC_50_: half maximum effective concentration; CC_50_: half maximum cytotoxic concentration; SI: Selectivity Index = ratio CC_50_/EC_50_; *Synthetic derivatives from lycorine

### Pretazettine and lycorine association against DENV-2 and ZIKV

**Fig. 1:**
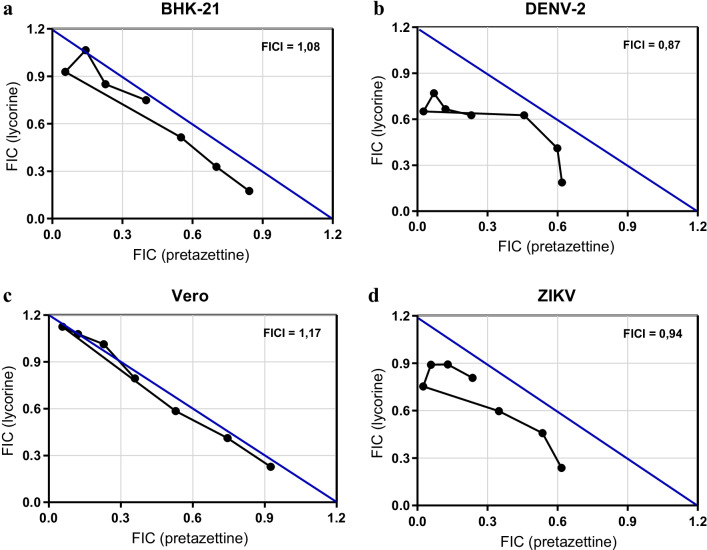
Isobolograms representing in vitro interactions of lycorine and pretazettine. The compounds were combined in fixed ration generating solutions which were tested for their cytotoxicity in BHK-21 (**a**) and Vero (**c**) cells and in parallel for their antiviral activity against DENV-2 (**b**) and ZIKV (**d**). The pair of doses were evaluated at the level of EC_50_ and CC_50_, which were determined by regression curves. Subsequently, their FIC was determined and plotted on the graphs above. All points on isobole represent dose pairs, FIC of pretazettine (x-axis) and lycorine (y-axis), that are expected to give the EC_50_ (in the case of viruses) or CC_50_ (in the case of cells). Plots are observed very close to the theoretical diagonal line, which means, in short, the indifferent effect of both compounds used together. The illustration strengthens the results obtained of statistical analyses, from which the fractional inhibitory concentration index (FICI) was obtained (shown at the upper right corner) and used to define interactions as indifferent for being between 0.5 and 4

Since pretazettine and lycorine were active against both viruses and could act by different mechanisms of action, the combinations at different proportions of these compounds were tested against DENV-2 and ZIKV to assess possible synergistic effects. Data analyses resulted in FICI values of 0.87 for DENV-2, 0.94 for ZIKV, 1.08 for BHK-21 cells, and 1.17 for Vero cells. According to Odds, 2003 [33], such interactions with 0.5 < FICI > 4.0, both in terms of antiviral activity and cytotoxicity, are considered indifferent. The absence of interactions between lycorine and pretazettine is most evident when the FIC was plotted and illustrated through the isobolograms. No synergistic or antagonistic interactions were identified (Fig. 1). Results are from two experiments performed with duplicates.

### Predicted pharmacokinetic and toxicity profiles

The pharmacokinetics and toxicity profiles, comprising 30 different ADMET properties, were calculated for lycorine, pretazettine, narciclasine, and NXP using the pkCSM platform. Kalbreclasine was also included, but its activity against ZIKV and DENV was not tested yet. The complete set of their predicted properties is available in Table 4. Pretazettine was predicted to have the highest intestinal absorption by both Caco2 cells permeability (log P_app_ > 0.90), which models the absorption of orally administered drugs, and by the intestinal absorption model (predicted to be 95% absorbed). The other compounds were predicted to have intermediate to low intestinal absorption. Pretazettine was also the molecule with the highest predicted steady-state volume of distribution (VDss) (VDss = 0.80), meaning better distribution in tissue rather than plasma, followed closely by lycorine (VDss = 0.79). Also, in terms of distribution properties, none of the molecules was predicted to readily cross the blood–brain barrier and penetrate the central nervous system, with kalbreclasine and NXP predicted to be very poorly distributed in the brain (log BB = -1.66 and -1.78, respectively). None of the compounds were predicted to be P450 inhibitors, and, instead, lycorine and pretazettine were predicted to be P450 substrates. In line with the predicted metabolism profiles of these two molecules were their excretion profiles. Both presented the highest total clearance (logCL = 1.03 for lycorine and logCL = 0.97 for pretazettine). In terms of their toxicity profile, none of the molecules were predicted to be mutagenic (AMES toxicity) or elicit skin sensitization. Three out of four were predicted to be hepatotoxic; however (NXP) was expected not to disrupt normal liver function. Three out of four molecules were also predicted to have low maximum recommended tolerated doses (MRTD), with only narciclasine presenting a high MRTD [0.60 log (mg/kg/day)] and NXP presenting an intermediate MRTD [0.34 log (mg/kg/day)].

**Table 4:** Predicted pharmacokinetic and toxicity profiles (ADMET) of the studied compounds

Compound/pkCSM property	LYC	PRZ	NCL	NXP	KBL
Water solubility	− 2.982	− 2.935	− 2.667	− 2.431	− 2.468
Caco2 permeability	0.517	1.192	0.586	− 0.47	− 0.417
Intestinal absorption (human)	68.852	95.146	68.248	47.934	35.249
Skin Permeability	− 3.319	− 3.810	− 2.931	− 2.749	− 2.736
P-Glycoprotein substrate	No	No	Yes	Yes	Yes
P-Glycoprotein I inhibitor	No	No	No	No	No
P-Glycoprotein II inhibitor	No	No	No	No	No
VDss (human)	0.790	0.802	− 0.561	− 0.540	− 0.537
Fraction unbound (human)	0.446	0.458	0.536	0.609	0.576
BBB permeability	− 0.153	− 0.084	− 1.315	− 1.784	− 1.660
CNS permeability	− 2.518	− 2.502	− 3.788	− 4.649	− 4.883
CYP2D6 substrate	Yes	No	No	No	No
CYP3A4 substrate	Yes	Yes	No	No	No
CYP1A2 inhibitor	No	No	No	No	No
CYP2C19 inhibitor	No	No	No	No	No
CYP2C9 inhibitor	No	No	No	No	No
CYP2D6 inhibitor	No	No	No	No	No
CYP3A4 inhibitor	No	No	No	No	No
Total Clearance	1.031	0.974	0.223	0.480	0.491
Renal OCT2 substrate	No	Yes	No	No	No
AMES toxicity	No	No	No	No	No
Max. tolerated dose (human)	− 0.386	− 0.566	0.597	0.335	0.181
hERG I inhibitor	No	No	No	No	No
hERG II inhibitor	No	No	No	No	No
Oral Rat Acute Toxicity (LD50)	2.674	2.417	1.982	2.854	2.621
Oral Rat Chronic Toxicity (LOAEL)	0.977	1.739	2.896	4.310	3.692
Hepatotoxicity	Yes	Yes	Yes	No	No
Skin Sensitisation	No	No	No	No	No
*T. pyriformis* toxicity	0.526	0.443	0.276	0.285	0.285
Minnow toxicity	1.877	2.583	4.660	7.014	8.766

LYC: lycorine; PRZ: pretazettine; NCL: narciclasine; NXP: narciclasine-4-*O*-*β*-D-xylopyranoside; \: kalbreclasine; ADMET: Absorption, Distribution, Metabolism, Excretion, and Toxicity

## Discussion

Numerous human viral diseases have a significant economic impact worldwide. The lack of licensed drugs to treat patients affected by many viral diseases such as dengue and Zika urges the need to discover, develop, and reposition drugs with innovative viral targets and mechanisms of action. In this work, in vitro assays were established and employed to identify extracts with antiviral properties against DENV and ZIKV and used to test thousands of extracts from different parts of hundreds of plant species from different Brazilian biomes.

Some of the active extracts showed virucidal properties since their activity relied on direct inactivation of the viral particles before virus adsorption. It is also speculated that compounds in the crude extracts could interfere with the virion envelope, masking the E and M proteins necessary for viral adsorption and entry into the host cells [42–45]. However, the composition and the precise mechanism of action of the virucidal extracts identified in this study needs further investigation.

One of our study's limitations is that some active non-virucidal extracts from *Annonaceae*, *Malpighiaceae,* and *Rubiaceae* families, when submitted to bioassay-guided fractionation, afforded no active fractions against DENV-2 and ZIKV. It is known that the phenomenon of synergistic effects of compounds present in an extract frequently causes loss-of-activity during bioassay-guided fractionation efforts, precluding identification or characterization of a relevant fraction for further evaluation, which is also a subject of further study. Therefore, we can infer that the antiviral activity verified in those extracts may have been due to the association of compounds separated in the fractionation steps or even their degradation along these procedures. Indeed, extracts and even their fractions may contain complex mixtures of substances which can result in a combination of different effects, not allowing reliable "go/no-go" decisions for further processing.

For those extracts presented active fractions against DENV-2 and ZIKV, we identified 18 known alkaloids and one flavonoid. However, few reports about the antiviral properties of these natural products were published. Only lycorine was detected in extracts of three species of *Hippeastrum*. Pseudolycorine was detected in *H. glaucescens* and *H. puniceum*. Among the 16 alkaloids identified, 12 were detected exclusively in *H. puniceum* and four in *H. glaucescens*. These results emphasize the importance of investigating this class of alkaloids as antivirals. Noteworthy, the alkaloids pretazettine, narciclasine, narciclasine-4-*O*-*β*-D-xylopyranoside, and the flavonoid rutine have never been tested against ZIKV. Just recently, the in vitro and in vivo antiviral properties of lycorine, found in more than 30 genera of plants, were reported against ZIKV [46]. Still, any lycorine derivatives have never been tested against this virus. Lycorine has broad-spectrum inhibitory activities against several RNA and DNA viruses, such as poliovirus [47], severe acute respiratory syndrome-associated coronavirus (SARS-CoV) [48], *Herpes simplex virus* (type 1) [49], DENV, *West Nile virus* and *Yellow fever virus* [50]. Some studies have put considerable effort into identifying related compounds or analogues from lycorine [30, 51, 52] by investigating its structure–activity relationships and analogues. These strategies have allowed us to discover promising less toxic molecules with higher or comparable levels of antiviral activity. Wang et al. [30] showed that a lycorine analogue, 1‐acetyl-lycorine, exhibited the most potent anti‐DENV activity with reduced cytotoxicity in A549 cells. In addition, 1-acetyl-lycorine inhibited hepatitis C virus (HCV) and suppressed the proliferation of multiple strains of Enterovirus 71 (EV71) through targeting viral proteases [53]. Here, we showed that 1-acetyl-lycorine and 1,2-diacetyl-lycorine have no antiviral activity against DENV-2 and a weak activity against ZIKV. We believe that different cell lines and assays could explain this discordance among those studies. Pretazettine, another alkaloid detected in active fractions of *Hippeastrum*, was isolated for the first time by Furusawa et al. [54] from *Narcissus tazetta*, an *Amaryllidaceae* species. Pretazettine strongly inhibited the activity of RNA-dependent DNA polymerase (reverse transcriptase) of the avian myeloblastosis virus [55]. Furthermore, this alkaloid exhibited consistent in vitro activity against Japanese encephalitis virus (JEV), YFV, Punta toro virus (PTV), Rift Valley fever virus [50], and human herpesvirus 1 (HHV-1) [49]. Interestingly, pretazettine inhibited the growth of the Rauscher virus and cellular protein synthesis in eukaryotic cells by a mechanism that does not affect DNA and RNA synthesis [50, 56–60].

In our work, we reasoned that the combination between pretazettine and lycorine could be more beneficial, given a possible synergistic action between them. This is supported by the fact that two compounds could act in different steps of the viral cycle and increase their effectiveness as antivirals. Indeed, combination therapies with multiple drugs have been demonstrated as effective approaches to treat several diseases such as malaria, tuberculosis, leishmaniasis, HIV, and HCV [61–67]. However, according to our results and the isobologram analyses, the antiviral effects of pretazettine-lycorine combinations in vitro showed to be indifferent against DENV-2 and ZIKV. The same was observed for their toxicity in Vero and BHK-21 cells.

Narciclasine, isolated from *H. puniceum* in this work, was reported to have selective and highly potent cytotoxic action on cancer cells, therefore being intensively investigated as an antitumor compound, in vitro and in vivo [68–72], as well as anti-inflammatory [73], anti-Alzheimer [74] and antiviral against JEV, YFV and DENV [50]. We have shown that narciclasine, though more toxic than lycorine and pretazettine was active against DENV-2 at a concentration 25 times less than presented by lycorine and 40 times less than presented by pretazettine. Similarly, the same was observed for ZIKV, being narciclasine activity verified at concentrations 45 times less than presented by lycorine and 95 times less than presented by pretazettine. Our data are similar to those reported by Gabrielsen et al. [50] for other flaviviruses as JEV, YFV, and DENV-4. Even though our study showed that minimum toxic doses and therapeutic doses were very similar, indicating a very narrow window for prophylaxis and therapy, there is room for improvement. As already mentioned, studies of the structure–activity relationship can be a promising area for exploring chemical radicals that can lead to increased antiviral activity and reduced cytotoxicity. Until now, the synthesis of narciclasine derivatives did not result in an optimized compound, at least concerning its antimitotic properties [75]. However, the discovery of new natural congeners of narciclasine with conserved antiviral activity can spur new studies of the antiviral activity of this molecule. Here we report the anti-DENV-2 and anti-ZIKV activity of narciclasine-4-*O*-*β*-D-xylopyranoside (NXP) isolated from bulbs of *H. puniceum*. NXP was 430 times less toxic than narciclasine in BHK-21 and Vero cells. Its EC_50_ values against DENV-2 and ZIKV were at concentrations 395 times those detected for narciclasine for both viruses. NXP was recently reported and characterized by Katoch et al. [41] in another species of Amaryllidaceae. It's relevant to mention that not all alkaloids identified in active extracts of *Hippeastrum* against DENV-2 and/ZIKV were tested in this work, and some of them, as narcissidine acetate, N-norlycoramine, 6-hidroxi-hippeastrine, lycoranine E, lycoranine C, crisarnine and acetyl-nerbowdine were not reported yet having any biological activity. The others, as pseudolycorine [76], pancratine and pancracine have already been shown to contain antiproliferative activities [77, 78]. Several studies indicate that alkaloids trigger a range of biological activities. Kalbreclasine, for example, exhibited a potent mitogenic action on splenic lymphocytes in healthy adult mice, stimulating their extensive proliferation [79, 80]. Nangustine has shown weak activities against trypanosome, leishmania, and plasmodium parasites [81]. Incartine [82–84] has shown significant neuroprotective effects against cell injury models in dopaminergic neuroblastoma SHSY5Y cells [85]. Lycoramine is a reversible cholinesterase inhibitor [86] and a modulator of nicotinic receptors, an important property in treating Alzheimer's and Parkinson's disease or neuroprotection against neurodegenerative disorders [87]. Nonetheless, these metabolites are isolated only in low amounts and therefore are not commercially available, representing an issue for thorough biological investigation.

Thankfully, in silico techniques have been introduced to drug discovery and development as tools to predict and optimize the ADMET properties of candidates at early stages [88]. These use physicochemical properties of compounds and advanced computational modelling to generate predicted models. Computational approaches may help minimize risks in following studies or taken important information for the decision to advance, hold or terminate a drug candidate [89]. Here, we used the pkCSM platform, which uses graph-based signatures to develop predictive models of central ADMET properties, [36] to analyse the compounds lycorine, pretazettine, narciclasine, NXP that exhibited some antiviral against DENV and ZIKV. ADMET properties of kalbreclasine were also analysed. The results supported these molecules as candidates for in vivo studies. Hopefully, chemical modifications studies can bring new derivatives that could be well optimized to present better bioavailability, oral absorption, clearance, volume of distribution, less toxicity, increased antiviral activity, and penetration into the central nervous system. Our findings corroborate the potential of plants to produce antiviral compounds. Indeed, there is still a lot of work to be done since we identified many  other compounds, not tested yet, in the active extracts reported by this study. A natural progression of this work is to assess the potential antiviral properties of all these compounds against ZIKV, DENV, and other viruses. Surely, innovative strategies are required to reveal and contribute the full range of chemical diversity of these valuable natural products to the antiviral drugs discovery process.

## Conclusions

The laborious screening of 7,000 plant extracts for antiviral activity led to the identification of extracts from the *Amaryllidaceae* family, which allowed the identification of several antiviral drug candidates. Our study supports the importance of exploring random crude extracts of plants to unveil new antiviral agents. We demonstrated the anti-ZIKV activity of pretazettine, narciclasine, and narciclasine-4-*O*-*β*-D-xylopyranoside. Finally, our findings indicate that plant species mainly of the genus *Hippeastrum* are a useful source of antiviral compounds against DENV-2 and ZIKV.

## Supplementary Information

**Additional file 1: Figure S1**. Map of the plates used for the validation assays of antiviral activity against DENV-2 and ZIKV using the MTT method. The validation was performed as per the High Throughput Screening (HTS) protocol described by Iversen et al., 2012 [24]. The model shows a combination of wells that produce the different intercalated signals, namely: uninfected cells (H), infected cells (L) and infected and treated cells (M), suitable for statistical analysis of absorbance readings of the product of the reduction of MTT by the cells. **Figure S2.** Validation of the antiviral HTS assay against DENV-2 and ZIKV using the MTT method. The validation was performed as per the High Throughput Screening (HTS) protocol described by Iversen et al., 2012 [24]. Raw data values of the plates on the day 3 (endpoint) of plate uniformity study with interleaved distribution of MIN (infected cells), MED (treated and infected cells) and MAX (cell control) signals analyzed by row (A and C) and by column (B and D). **Figure S3.** CC_50_ and EC_50_ titration curves of pretazettine (PTZ),lycorine (LYC), narciclasine (NCL), and narciclasine-4-*O*-*β*-*D*-xylopiranoside (NXP) against DENV-2 and ZIKV. The values were determined by regression curve using GraphPad Prism 5 based on nonlinear logistic regression of the dose-response curves. The values correspond to the average and standard deviation of three independent assays with at least 8 concentrations of the substance. The red dots correspond to the concentration at which the substance has reached host cell toxicity in antiviral assays.

## Data Availability

All data generated or analysed during this study are included in this published article.

## Abbreviations

ADMET: Stands for Absorption, Distribution, Metabolism, Excretion and Toxicity
ATCC: American Type Culture Collection
CAPES: Coordenação de Aperfeiçoamento de Pessoal de Nível Superior
CC_50_: Half maximum cytotoxic concentration
CNPq: Conselho Nacional de Desenvolvimento Científico e Tecnológico
CPE: Cytopathic effect
DENV: Dengue virus
DENV-2: Dengue virus serotype 2
DENV-4: Dengue virus serotype 4
DMSO: Dimethyl sulfoxide
EC_50_: Half maximum effective concentration
EDTA: Ethylenediamine tetraacetic acid
FBS: Fetal bovine serum
FIC: Fractional inhibitory concentration
FICI: Fractional inhibitory concentration index
*H. puniceum*: *Hippeastrum puniceum*
*H. glaucescens*: *Hippeastrum glaucescens*
HCV: Hepatitis C virus
HHV-1: Human herpesvirus 1
HIV: Human Immunodeficiency virus
HRMS/MS: High resolution mass spectrometry in tandem with mass spectrometry
JEV: Japanese encephalitis virus
MEM: Minimum Eagle's Medium
m.o.i.: Multiplicity of infection
MTT: 3-(4,5-Dimethylthiazol-2-yl)-2,5-diphenyl tetrazolium bromide
MSn: Mass spectrometry
MRTD: Maximum recommended tolerated doses
NMR: Nuclear magnetic resonance
NXP: Narciclasine-4-*O*-*β*-D-xylopyranoside
PFU: Plaque forming unit
p.i.: Post infection
UHPLC: Ultra high pressure liquid chromatography
YFV: Yellow fever virus
ZIKV: Zika virus
