# Supplementary material for: Searching for plant-derived antivirals against dengue virus and Zika virus
Source: Virol J. 2022 Feb 22;19:31. doi: 10.1186/s12985-022-01751-z (PMC8861615; doi:10.1186/s12985-022-01751-z)
Supplement: Supplementary file 1 — Additional file 1: Figure S1. Map of the plates used for the validation assays of antiviral activity against DENV-2 and ZIKV using the MTT method. The validation was performed as per the High Throughput Screening (HTS) protocol described by Iversen et al., 2012 [24]. The model shows a combination of wells that produce the different intercalated signals, namely: uninfected cells (H), infected cells (L) and infected and treated cells (M), suitable for statistical analysis of absorbance readings of the product of the reduction of MTT by the cells. Figure S2. Validation of the antiviral HTS assay against DENV-2 and ZIKV using the MTT method. The validation was performed as per the High Throughput Screening (HTS) protocol described by Iversen et al., 2012 [24]. Raw data values of the plates on the day 3 (endpoint) of plate uniformity study with interleaved distribution of MIN (infected cells), MED (treated and infected cells) and MAX (cell control) signals analyzed by row (A and C) and by column (B and D). Figure S3. CC50 and EC50 titration curves of pretazettine (PTZ),lycorine (LYC), narciclasine (NCL), and narciclasine-4-O-β-D-xylopiranoside (NXP) against DENV-2 and ZIKV. The values were determined by regression curve using GraphPad Prism 5 based on nonlinear logistic regression of the dose-response curves. The values correspond to the average and standard deviation of three independent assays with at least 8 concentrations of the substance. The red dots correspond to the concentration at which the substance has reached host cell toxicity in antiviral assays. [file 12985_2022_1751_MOESM1_ESM.docx]

| **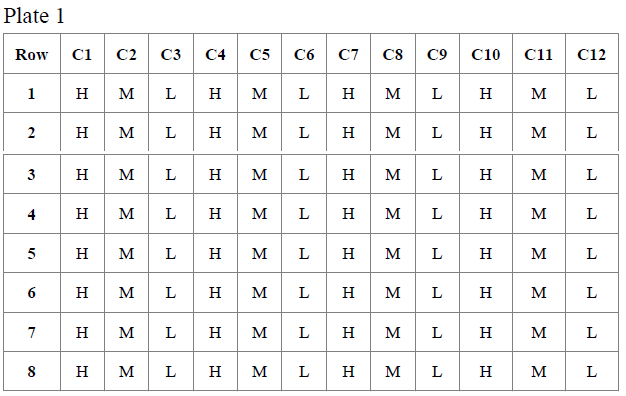** | **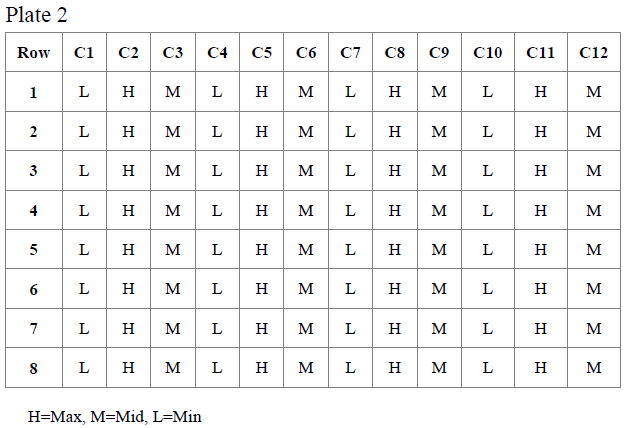** |
| --- | --- |
| 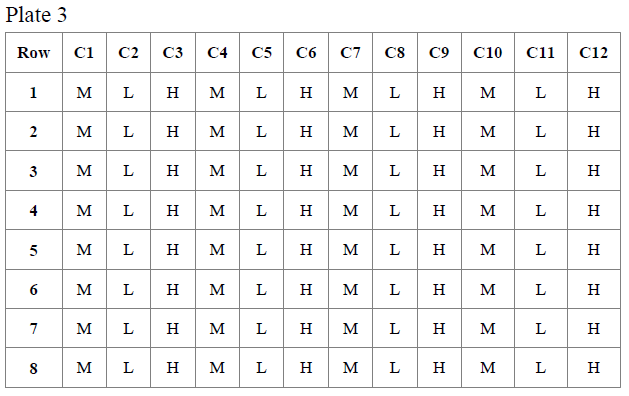 | |

**Figure S1- Map of the plates used for the validation assays of antiviral activity against DENV-2 and ZIKV using the MTT method**. The validation was performed as per the High Throughput Screening (HTS) assay manual described by Iversen et al., 2012 [24]. The model shows a combination of wells that produce the different intercalated signals, namely: uninfected cells (H), infected cells (L) and infected and treated cells (M), suitable for statistical analysis of absorbance readings of the product of the reduction of MTT by the cells.

1. **DENV-2 B- DENV-2**

1. **ZIKV D- ZIKV**

**Figure S2 - Validation of the antiviral HTS assay against DENV-2 and ZIKV using the MTT method.** The validation was performed as per the High Throughput Screening (HTS) protocol described by Iversen et al., 2012 [24]. Raw data values of the plates on the day 3 (endpoint) of plate uniformity study with interleaved distribution of MIN (infected cells), MED (treated and infected cells) and MAX (cell control) signals analyzed by row (A and C) and by column (B and D).

|  |  |  | |  | |  |
| --- | --- | --- | --- | --- | --- | --- |
|  |  |  | |  | |  |
|  |  |  | |  | |  |
|  |  | |  | |  | |

**Figure S3 CC_50_ and EC_50_ titration curves of pretazettine (PTZ) lycorine (LYC), narciclasine (NCL) and narciclasine-4-*O*-*β*-D-xylopiranoside (NXP) against DENV-2 and ZIKV.** The values were determined by regression curve using GraphPad Prism 5 based on logistic non-linear regression of the dose-response curves. The values correspond to the average and standard deviation of three independent assays with at least 8 concentrations of the substance. The red dots correspond to the concentration at which the substance has reached host cell toxicity in antiviral assays.
